# Cardiac Morphology, Function, and Left Ventricular Geometric Pattern in Patients with Hypertensive Crisis: A Cardiovascular Magnetic Resonance-Based Study

**DOI:** 10.3390/jcdd10090367

**Published:** 2023-08-27

**Authors:** Mohammed A. Talle, Anton F. Doubell, Pieter-Paul S. Robbertse, Sa’ad Lahri, Philip G. Herbst

**Affiliations:** 1 Division of Cardiology, Department of Medicine, Faculty of Medicine and Health Sciences, Stellenbosch University and Tygerberg Hospital, Cape Town 7505, South Africa; 2 Department of Medicine, Faculty of Clinical Sciences, College of Medical Sciences, University of Maiduguri and University of Maiduguri Teaching Hospital, Maiduguri 600004, Nigeria; 3 Division of Emergency Medicine, Department of Medicine, Faculty of Medicine and Health Sciences, Stellenbosch University and Tygerberg Hospital, Cape Town 7505, South Africa

**Keywords:** hypertensive crisis, hypertensive emergency, hypertensive urgency, cardiovascular magnetic resonance, cardiac morphology and function, geometric patterns, late gadolinium enhancement

## Abstract

(1) Background: Altered cardiac morphology and function are associated with increased risks of adverse cardiac events in hypertension. Our study aimed to assess left ventricular (LV) morphology, geometry, and function using cardiovascular magnetic resonance (CMR) imaging in patients with hypertensive crisis. (2) Methods: Patients with hypertensive crisis underwent CMR imaging at 1.5 Tesla to assess cardiac volume, mass, function, and contrasted study. Left ventricular (LV) function and geometry were defined according to the guideline recommendations. Late gadolinium enhancement (LGE) was qualitatively assessed and classified into ischemic and nonischemic patterns. Predictors of LGE was determined using regression analysis. (3) Results: Eighty-two patients with hypertensive crisis (aged 48.5 ± 13.4 years, and 57% males) underwent CMR imaging. Of these patients, seventy-eight percent were hypertensive emergency and twenty-two percent were urgency. Diastolic blood pressure was higher under hypertensive emergency (*p* = 0.032). Seventy-nine percent (92% of emergency vs. 59% of urgency, respectively; *p* = 0.003) had left ventricular hypertrophy (LVH). The most prevalent LV geometry was concentric hypertrophy (52%). Asymmetric LVH occurred in 13 (22%) of the participants after excluding ischemic LGE. Impaired systolic function occurred in 46% of patients, and predominantly involved hypertensive emergency. Nonischemic LGE occurred in 75% of contrasted studies (67.2% in emergency versus 44.4% in urgency, respectively; *p* < 0.001). Creatinine and LV mass were independently associated with nonischemic LGE. (5) Conclusion: LVH, altered geometry, asymmetric LVH, impaired LV systolic function, and LGE are common under hypertensive crisis. LVH and LGE more commonly occurred under hypertensive emergency. Longitudinal studies are required to determine the prognostic implications of asymmetric LVH and LGE in hypertensive crisis.

## 1. Introduction

Despite the progress made in the evaluation and management of systemic hypertension, the incidence of hypertensive crisis remains disproportionately high in sub-Saharan Africa [1]. Hypertensive crisis is defined as an acute, severe blood pressure rise to ≥180 mmHg systolic, and/or ≥110 mmHg diastolic, and constitutes up to 25% of all medical emergencies [2,3]. Hypertensive crisis with associated acute target organ damage is considered a hypertensive emergency; hypertensive urgency occurs where there is no evidence of acute target organ damage [2]. In patients with hypertensive emergency, acute hypertension-mediated organ damage (HMOD) commonly involves the cardiovascular system, and includes acute pulmonary edema/heart failure, acute coronary syndrome, and, less commonly, acute aortic dissection [4].

Structural and functional left ventricular (LV) remodeling, including left ventricular hypertrophy (LVH), altered LV geometry, diastolic dysfunction, and systolic dysfunction, represent the cardinal features of hypertensive heart disease [HHD] [5]. In patients with hypertensive crisis, LVH is often disproportionately more severe compared to hypertension without episodes of crisis, especially under black hypertensive patients where LV wall thickness can reach up to 20 mm [6]. Similarly, asymmetric LV wall thickening and hypertrophy (not uncommon in patients with more severe degrees of LVH) presents a diagnostic challenge in differentiating HHD from other causes of LVH, especially hypertrophic cardiomyopathy (HCM). Apart from the challenges in differentiating disproportionate hypertensive LVH from other non-hypertensive phenocopies, LVH and abnormal LV geometry have been found to be significantly associated with increased morbidity and mortality in patients with hypertension [5,7]. Additionally, LVH and HHD concurrently develops with myocardial fibrosis (including both interstitial fibrosis and focal replacement fibrosis), and these changes provide a substrate for ventricular arrhythmias and sudden cardiac death [8,9,10,11]. Other factors associated with increased risks of major adverse cardiovascular events (MACE) in hypertensive patients include systolic and diastolic dysfunction [12,13]. In general, LV systolic function is preserved in patients with hypertensive crisis, and the symptoms in patients presenting with acute pulmonary edema and heart failure is mainly driven through exacerbation of diastolic dysfunction [14]. However, global longitudinal strain measured with echocardiography has revealed impaired systolic function in patients with hypertensive emergency, suggesting the presence of a sub-clinical systolic dysfunction [15].

Although structural and functional cardiac alterations, including late gadolinium enhancement (LGE) on cardiovascular magnetic resonance (CMR) imaging, have been associated with an increased risk of MACE in patients with hypertension, this has not been systematically studied in patients with hypertensive crisis. Cardiac magnetic resonance imaging is currently the gold standard for evaluating cardiac volumes, mass, and function, surpassing echocardiography, the primary diagnostic tool in the past. In addition, CMR imaging allows for myocardial tissue characterization using contrasted and parametric mapping sequences, assisting with the etiologic diagnosis of various forms of cardiomyopathy. Given the proven utility in patients with LVH [16], CMR imaging can be employed in persons with hypertensive crisis, especially in the presence of disproportionate LVH, asymmetric thickening, and left ventricular wall thicknesses greater than the conventional threshold of <15 mm for HHD [6,17].

Considering the prognostic implications of altered LV morphology and function in hypertension, and the lack of CMR-based studies, we set out to study the cardiac morphology, function, and left ventricular geometric patterns using CMR imaging on patients with a hypertensive crisis. Furthermore, we sought to compare the findings obtained under hypertensive emergency with hypertensive urgency. We assessed myocardial fibrosis using LGE, a marker of poor prognosis in various cardiac diseases, and compared findings under hypertensive emergency with hypertensive urgency.

## 2. Materials and Methods

### 2.1. Study Population

We carried out an observational study in patients aged 18 years and above with hypertensive crisis, referred to Tygerberg Hospital in the Western Cape province of South Africa. The diagnosis of hypertensive crisis was based on a systolic blood pressure (BP) of ≥180 mmHg, and/or a diastolic BP of ≥110 mmHg. Patients were further classified as having hypertensive urgency where there was no evidence of acute HMOD, and hypertensive emergency when patients presented with acute HMOD [2,18]. Hypertensive emergency was categorized into acute pulmonary edema, myocardial infarction, and neurological emergencies based on the type of acute HMOD.

Guideline-directed investigations, including high-sensitive cardiac troponin T (hs cTnT) and N-terminal prohormone of brain natriuretic peptide (NT-proBNP), were carried out in all participants. Myocardial infarction was defined using the fourth universal definition [19], and assessment of the coronary arteries was undertaken as per clinical indication and determined by the managing cardiologist. All patients with neurological emergencies had computed tomography (CT) imaging of the brain as part of their standard care. Patients with hypertensive disorders of pregnancy, patients with an altered level of consciousness, and those who declined their consent to participate were excluded from the study. The study was approved by the Health Research Ethics Committee (HREC) of Stellenbosch University and all participants granted written consent. The Declaration of Helsinki was adhered to.

### 2.2. Cardiac Magnetic Resonance Imaging Acquisition and Analysis

CMR imaging was undertaken in all participants using a 1.5 T scanner (Magnetom Avanto, Siemens Healthcare, Erlangen, Germany) within 48 h of presentation to Tygerberg Hospital. Breath-held, steady-state free precession short-axis cine images covering the heart from the base to the apex was obtained for the assessment of the ventricular volume, mass, and function [20]. Late gadolinium enhancement images were obtained using inversion-recovery fast gradient-echo sequences after 10–12 min of intravenous administration of 0.2 mL/kg gadolinium-based contrast agent (Gadovist, Bayer Pharma AG 51368, Leverkusen, Germany). The inversion time for nulling the myocardium was optimized in each participant using the TI-scout sequence. Images were obtained in two orthogonal planes, including the short and standard long-axis sections.

CMR data was analyzed by M.A.T. using a commercially available software (CVI^42^; Circular cardiovascular Imaging, version 5.13.10 [2678] Calgary, Canada). Epicardial and endocardial contours were drawn under the end-diastolic and end-systolic phases to determine the end-diastolic volume (EDV), end-systolic volume (ESV), stroke volume (SV), and LV mass excluding the papillary muscle from the blood pool. The biplane area-length method was used to determine the left atrial volume (LAV), and the LAV index was derived from dividing the LAV by the body surface area (BSA). The presence and location of LGE was visually assessed and then classified into ischemic (subendocardial or transmural LGE in a coronary distribution) and nonischemic LGE (patchy, mid-wall, focal or right ventricular [RV] insertion point LGE) [21]. Areas of LGE extending from the pedicle of the right ventricle through a rounded contour into the LV myocardium was reported as RV insertion point fibrosis [22].

### 2.3. Left Ventricular Hypertrophy and Geometric Patterns

The indexed LV mass and the LV EDV above the 95^th^ percentile of the age- and gender-specific CMR reference range were used to define LVH and LV dilatation [23]. The relative wall mass (RWM), the conceptual equivalent of relative wall thickness, was obtained through dividing the LV mass by the LV EDV, and an increased RWM was defined as age- and gender-specific values above the 95^th^ percentile of the reference value [24]. Maximum LV wall thickness was measured using end-diastolic short-axis cine views of the LV, excluding ventricular (RV and LV) trabeculations. Asymmetric wall thickening was defined as an end-diastolic wall thickness of >15 mm in at least one LV segment that was >1.5 times the thickness of the opposing segment, and LV geometric patterns were defined as follows [25,26]: (1) Normal geometry—normal indexed LV mass, volume, and RWM; (2) concentric remodeling—normal indexed LV mass, reduced indexed LV EDV, and increased RWM; (3) concentric hypertrophy—increased indexed LV mass, increased RWM, and normal indexed LV EDV; and (4) eccentric hypertrophy—increased indexed LV mass and volume with normal RWM. Asymmetric LVH was defined as an increased indexed LV mass with asymmetric wall thickening. Participants with subendocardial LGE consistent with myocardial infarction and subepicardial LGE (consistent with myocarditis) were excluded from the assessment of asymmetric wall thickening and asymmetric LVH.

### 2.4. Statistical Analysis

The sample size was estimated a priori, leveraging on the accuracy and excellent reproducibility of CMR imaging [27]. Using a standard deviation of 10 for the indexed LV mass in HHD [17], twenty-two participants with hypertensive crisis will be required to detect a difference of 15g in LV mass indexed to their BSA at an alpha level of 0.05 with a power of 90%. Data were analyzed using SPSS version 28 (SPSS Inc, Chicago, IL, USA). The Shapiro–Wilk test was used to assess the normality of the variables under study. Continuous variables were presented as mean ± SD or median [IQR] and compared using the Student’s *t*-test, Mann–Whitney U test, and one-way ANOVA with post-hoc analysis, as appropriate. Categorical variables were presented as proportions and percentages and compared using Fisher’s exact test. The correlation of continuous variables was determined using Spearman’s correlation (two-tailed *p*-value). Binary regression analysis was used to determine factors associated with nonischemic LGE in a univariate model. Variables that demonstrated a significant association with LGE were included in a multivariate model to determine their independent contribution. GraphPad Prism version 6.04 for MacOS (GraphPad Software 10.0.0 (131), www.graphpad.com (accessed on 12 July 2023)) was used in making scatter plots. A two-tailed *p* value of <0.05 was considered statistically significant for all analyses.

## 3. Results

### 3.1. Clinical and Demographic Characteristics

**Figure 1:**
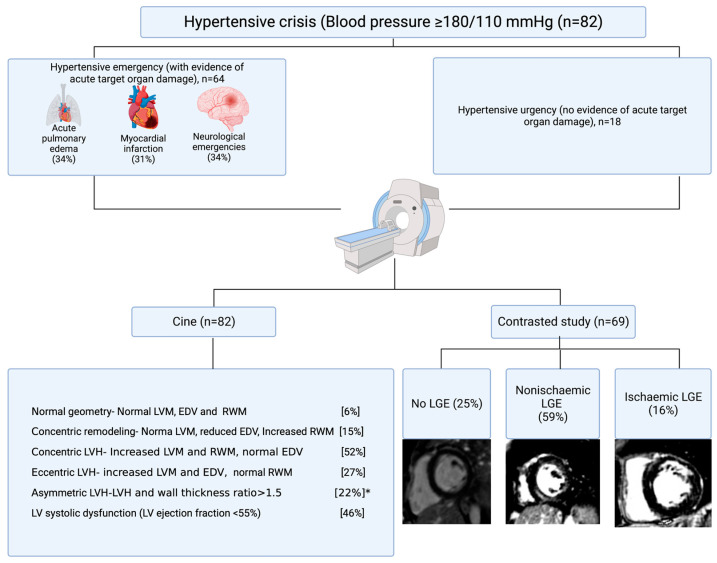
Study flow chart. Abbreviations: EDV, end-diastolic volume (indexed); LGE, late gadolinium enhancement; LV, left ventricular; LVH, left ventricular hypertrophy; LVM, left ventricular mass (indexed); and RWM, relative wall mass. * Prevalence of asymmetric LVH in participants without ischemic LGE (cuts across both concentric and eccentric LVH).

Eighty-two participants (Figure 1) with hypertensive crisis (mean age 48.5 ± 13.4 years, and 57% men), comprising 64 (78%) with hypertensive emergency and 18 (22%) with hypertensive urgency, underwent CMR imaging to assess their cardiac morphology, function, and LV geometry. The ages of the patients assessed were similar under hypertensive emergency and urgency (*p* = 0.972). Forty-two (66%) of the patients with hypertensive emergency were men. The diastolic blood pressure (*p* = 0.032), creatinine (*p* < 0.001), hs cTnT (*p* = 0.001), and NT-proBNP (*p* = 0.012) were all higher under hypertensive emergency (Table 1). Two thirds of the acute HMOD were cardiovascular, comprising 22 (34%) cases of acute pulmonary edema and 20 (31%) myocardial infarctions; meanwhile, the composite of neurological emergencies (intracranial hemorrhage, ischemic stroke, transient ischemic attack, and hypertensive encephalopathy) constituted the remaining 22 (34%) cases.

### 3.2. Cardiac Morphology and Function

The findings that were obtained through CMR imaging are summarized in Table 1. Extracardiac findings included adrenal mass (biochemically confirmed to be phaeochromocytoma in two participants), pulmonary tuberculosis (two participants), and diaphragmatic hernia (one participant).

#### 3.2.1. Ventricular and Atrial Volumes and Functions

Indexed LV EDVs and LV ESVs were increased in 21 (26%) and 36 (44%) of the hypertensive crisis cohort and were significantly higher in hypertensive emergency than hypertensive urgency (*p* = 0.001 and *p* < 0.001, respectively; Table 1). Thirty-seven (46%) of the participants with hypertensive crisis had impaired LV systolic function, and this occurred exclusively in the hypertensive emergency group. The left atrial volume was higher in patients under hypertensive emergency compared to urgency (*p* = 0.007). Moreover, the indexed LV EDVs and LV ESVs were higher in acute pulmonary edema compared to neurological emergencies (*p* < 0.05 for both). In contrast, indexed LV EDVs were found to be similar in patients with acute pulmonary edema and myocardial infarction (*p* = 0.106); indexed LV ESVs were found to be higher in acute pulmonary edema (*p* = 0.013). The indexed LA volume was found to be 12 mL/m^2^ higher in the hypertensive emergency group (*p* = 0.004). Comparison of the CMR findings in the subtypes of hypertensive emergency has been illustrated in Supplementary Table S1.

**Figure 2:**
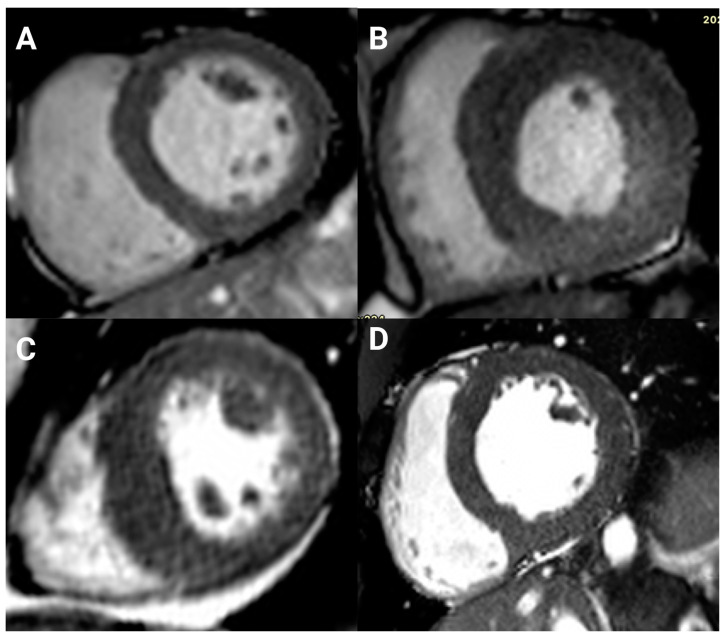
Different LV geometric patterns. (**A**) Normal geometry; (**B**) concentric hypertrophy; (**C**) asymmetric hypertrophy; and (**D**) eccentric hypertrophy.

Maximum wall thicknesses of >15 mm and ≥20 mm occurred in forty (49%) and nine (11%) participants, respectively. Excluding patients with infarct-pattern LGE, 28 (48%) participants with a contrasted study had a maximum wall thickness of >15 mm, and mainly involved the basal to mid anterior septum and the mid inferior septum. Asymmetric LVH occurred in 13 (22%) of the hypertensive crisis patients without ischemic LGE and constituted 20% of all LVH in the entire cohort. The absolute and indexed LV masses were higher by 89 g (*p* = 0.014) and 21 g/m^2^ (*p* = 0.019) in the asymmetric LVH group (Supplementary Table S2). Univariate logistic regression revealed a significant association between asymmetric LVH with left ventricular mass (*p* = 0.009), indexed left ventricular mass (*p* = 0.014), LV mass/volume ratio (*p* < 0.001), and maximum wall thickness (*p* < 0.001). However, only maximum wall thickness maintained an independent association with asymmetric LVH (OR 2.9 {95%CI, 1.47–5.76}, *p* = 0.002, Nagelkerke R Square 0.644) in multivariate analysis. Exemplar CMR images of LV geometric patterns are presented in Figure 2.

#### 3.2.2. Left Ventricular Mass, Left Ventricular Hypertrophy, Geometric Pattern, and Maximum Wall Thickness

The absolute and indexed LV masses were higher in patients with hypertensive emergency compared with urgency (*p* < 0.001, Table 1). Sixty-five (79%) patients with hypertensive crisis had LVH, and this was significantly higher under hypertensive emergency than urgency (88% vs. 50%, respectively; *p* = 0.004). Concentric LVH was the most prevalent geometric pattern and occurred in 52% of all the hypertensive crisis cohort, followed by eccentric hypertrophy in 27% (Table 2). Normal geometry (*p* = 0.034) and concentric remodeling (*p* = 0.011) were more prevalent in the group with hypertensive urgency. Clinical, biochemical, and CMR findings in patients with and without LVH are illustrated in Supplementary Table S3.

### 3.3. Late Gadolinium Enhancement

**Figure 3:**
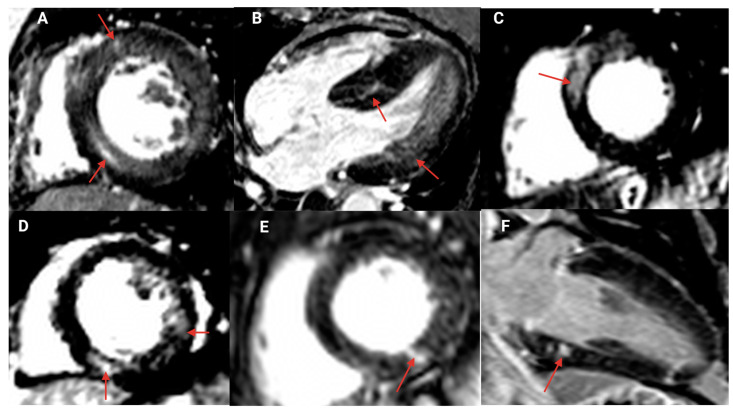
Different patterns of nonischemic late gadolinium enhancement (arrows) in patients with hypertensive crisis. Basal and mid ventricular short axis (**A**,**C**–**E**); horizontal long-axis view (**B**) showing septal hypertrophy with mid myocardial late gadolinium enhancement in inferior septum and lateral wall of the left ventricle; vertical long-axis view (**F**) showing areas of focal late gadolinium enhancement in basal and apical inferior wall of the left ventricle.

Gadolinium-based contrast agent (Gadovist^®^) was administered to 69 (84%) of all participants (all hypertensive urgency, and 80% of hypertensive emergency). The contrast agent was not administered to 13 patients (16%) due to renal impairments. Fifty-two individuals (75%) of the group receiving the contrast agent (86% of hypertensive emergency, and 44% of hypertensive urgency, respectively; *p* < 0.001) demonstrated LGE (Table 3). Nonischemic LGE (Figure 3) occurred in 41 patients (59%), while 11 patients (16%) had subendocardial/transmural LGE consistent with myocardial infarction. Two of the infarct-pattern LGE were consistent with old myocardial infarction, while 78% of patients with acute myocardial infarction and infarct-pattern LGE had microvascular obstructions. Patients with nonischemic LGE (Supplementary Table S4) displayed elevated creatinine (*p* < 0.001), hs cTnT (*p* = 0.002), NT-proBNP (*p* = 0.002), indexed LV mass (*p* < 0.001), indexed LA volume (*p* = 0.003), and prevalence of LVH (*p* < 0.001). Nonischemic LGE was significantly associated with creatinine, LV mass, indexed LV mass, maximum LV wall thickness, LV mass/volume, and indexed LA volume in a univariate regression analysis. However, only creatinine (*p* = 0.022) and the LV mass (*p* = 0.016) were significantly associated with nonischemic LGE on multivariate analysis (Nagelkerke R Square, 0.54, Table 4).

### 3.4. Intra- and Inter-Reader Reliability for Quantitative Measurements

We have previously published our inter-observer variability for quantitative cardiac parameters [28]. MAT performed a re-read of 15 randomly selected cases with the following correlations: LV EDV (ICC = 0.95), LV ESV (ICC = 0.95), LV SV (ICC = 0.92), LV mass (ICC = 0.96), LV ejection fraction (0.90), RV EDV (ICC = 0.85), and LA volume (0.95). The *p*-value was <0.001 for all correlations.

## 4. Discussion

Hypertensive crisis typically occurs in the setting of uncontrolled hypertension with underlying LVH. Using CMR imaging, we have demonstrated the following: (1) Seventy-nine percent of all hypertensive crisis patients had LVH, which was more frequent under hypertensive emergency (88%) compared to hypertensive urgency, where 50% of the group had LVH. (2) Asymmetric LVH occurred in 22% of participants after excluding those with infarct-pattern LGE. (3) Forty-six percent of all participants with hypertensive crisis had an impaired LV systolic function, and this mainly occurred in the group with the hypertensive emergency. (4) Nonischemic LGE occurred in 59% of all participants with hypertensive crisis that received the contrast agent.

The prevalence of LVH in hypertensive emergency in our study is comparable to what was reported by Rubin et al. in their cohort of malignant hypertension [29]. However, the ERIDANO study reported LVH in 34% of their cohort with hypertensive crisis [30]. This discrepancy may be related to differences in the study populations. Furthermore, 78% of our cohort had hypertensive emergency, while 85% of participants in the ERIDANO study had hypertensive urgency. Secondly, blood pressures were higher in our hypertensive emergency group when compared with the ERIDANO cohort. Left ventricular mass has been reported to be disproportionately more severe in patients with hypertensive crisis when compared to hypertension without episodes of crisis. In one study, Gosse et al. reported their indexed LV mass to be 58% higher than the upper limit of normal in patients with malignant hypertension [15]. A strong and consistent association has been demonstrated between an increased LV mass index and cardiovascular mortality in hypertension [31,32], suggesting that the increased LV mass and high prevalence of LVH observed in our cohort may partly contribute towards a poor prognosis in the group with hypertensive emergency compared to hypertensive urgency.

We found concentric LVH to be the most common geometric pattern occurring in hypertensive urgency and emergency. Although this is similar to other studies on hypertensive LV geometric patterns, there is a lack of data on LV geometric patterns in patients with hypertensive crisis [33,34]. Most studies on LV geometry in hypertension were carried out using echocardiography, an important caveat to be considered when comparing findings from different imaging modalities. Nonetheless, concentric remodeling, representing stage A disease (the earliest form of HHD) in a recently proposed staging for HHD, has been associated with an increased risk of incident coronary artery disease and stroke [5,33]. Similarly, concentric LVH, the most prevalent LV geometry in our study, as well as eccentric LVH, have been associated with poor cardiovascular outcomes [7].

Generally, LV systolic function, determined via an ejection fraction, is preserved in patients with HHD and hypertensive crisis, and only begins to deteriorate under late-stage disease [5,14]. We found impaired LV systolic function in 46% of all hypertensive crises that mainly involved patients with hypertensive emergency, which is higher than what was reported in other studies [29,30]. However, Gosse et al. reported impaired systolic function using global longitudinal strain measured via echocardiography in 53% of patients with acute phase malignant hypertension [15]. The apparent preservation of left ventricular systolic function measured using an ejection fraction in HHD may be related to concentric hypertrophy, preservation of radial function, and the reduction in size of the LV cavity. Regardless of methodology, impaired systolic function increases mortality in patients with hypertension [5]. A possible explanation for the high prevalence of systolic dysfunction in our cohort includes a late presentation with HHD and an increased prevalence of renal impairment. However, we do not have specific data on this, and dedicated research will be required to answer this question. Additionally, the high prevalence of coronary artery disease reported in patients presenting with hypertensive crisis may contribute to systolic dysfunction [35]. Ischemic LGE constituted 16% of all LGE and occurred in 55% of the group with myocardial infarction.

A maximum wall thickness >15 mm and asymmetric LVH occurred in 46% and 22% of our cohort with nonischemic LGE, respectively. Reports of asymmetric hypertensive LVH emerged more than three decades ago [36,37], and this is being increasingly corroborated, especially with the advent of CMR. In the Bristol study, Rodrigues et al. reported asymmetric LVH in 21% of patients with HHD [17]. While the Bristol cohort involved patients diagnosed with HHD and referred them for CMR imaging to differentiate HHD from HCM, our cohort consisted of all patients referred with hypertensive crisis. Nonetheless, we found similar indexed LV masses and maximum wall thicknesses to what was reported in the Bristol study (Table 2). The pathogenetic mechanism involved in hypertensive asymmetric wall thickening is not fully understood. A proof of concept for the upregulation of the renin-angiotensin-aldosterone pathway, a common phenomenon in hypertensive crisis, has been demonstrated [38]. Other factors include dense sympathetic innervation of the basal septum and duration of hypertension [39].

CMR imaging is the gold standard for assessing myocardial fibrosis, manifesting as areas of LGE in replacement fibrosis. However, there are no data on contrast CMR studies in patients with hypertensive crisis. We found LGE in 75% of contrasted studies (59% nonischemic LGE), exceeding what was reported in other studies involving hypertensive patients. Rudolf et al. reported nonischemic LGE in 50% of patients with arterial hypertension, while Treibel et al. reported a prevalence of 28% [40,41]. In the REMODEL study involving asymptomatic hypertensive patients, Iyer et al. only found nonischemic LGE in 18% of patients [10]. The prevalence of nonischemic LGE observed in our cohort is in the range reported among patients with HCM [42]. This has an important clinical implication when taken in the context of the prognostic implications of LGE. Nonischemic LGE demonstrated an independent association with the composite of acute coronary syndrome, heart failure hospitalization, stroke, and cardiovascular mortality in the REMODEL study [10].

Isolated RV insertion point LGE, defined using an objective criterion [22], constituted about a quarter of all LGE in our cohort. In general, RV insertion point fibrosis has been attributed to the impact of circumferential and longitudinal forces associated with systolic shortening of the left and right ventricles, respectively [43]. A high prevalence of RVIP fibrosis with prognostic implications has been reported in idiopathic pulmonary hypertension and was attributed to right ventricular hypertrophy [44]. Mikami et al. also reported an increased risk of heart failure admission and mortality in patients with dilated cardiomyopathy and isolated RVIP fibrosis [22]. However, the relationship between altered LV geometric patterns and contractility with RV insertion point fibrosis under hypertension and hypertensive crisis has not been determined. The high prevalence of insertion point fibrosis (isolated and in combination with mid-wall fibrosis) demonstrated in our cohort warrants an outcome study to determine its prognostic implications in patients with hypertensive crisis.

The findings from our study have important clinical implications, especially when viewed in the context of the increased risk of cardiovascular morbidity and mortality associated with hypertensive crisis. The increased prevalence of altered structures and functions observed in the hypertensive emergency group may be associated with the increased risks of MACEs compared to hypertensive urgency. Similarly, a higher prevalence of LGE compared to studies involving hypertension without crisis [10,11] suggests a more advanced cardiac remodeling and poor prognosis. These will help in risk stratification and prognostication in patients presenting with a hypertensive crisis. Although CMR imaging is not currently indicated in the routine evaluation of patients with systemic hypertension, the high prevalence of asymmetric LVH, impaired systolic function, and LGE observed among the hypertensive crisis cohort warrants lowering the threshold for CMR in these patients, as an improved assessment with CMR imaging may lead to more accurate assessments to ultimately enhance patient care. Furthermore, CMR imaging has the advantage of identifying secondary etiologies of hypertension, including adrenal mass, renal artery stenosis, and coarctation of the aorta, some of which are associated with an increased risk of hypertensive crisis.

### Study Limitations

Our study has important limitations. Impaired renal function precluded some of the participants from undergoing a contrast MRI. Given the interconnectedness of hypertensive crisis and renal impairment, it is not feasible to exclude such patients from this study. Parametric T1 mapping and CMR-FT strain, which have previously been demonstrated to be useful in differentiating HHD from HCM and other phenocopies, including cardiac amyloidosis, were not included. However, gadolinium kinetics was normal in all the contrasted studies, making cardiac amyloidosis unlikely.

Quantification of LGE is becoming increasingly recognized in the risk stratification of patients with cardiovascular diseases. Given the extent of LGE in the cohort, it will be interesting to see how quantitative assessment of LGE will aid risk classification and prognosis in patients with hypertensive crisis. This was not performed in this study.

A known limitation of LGE is its limited ability to detect diffuse myocardial fibrosis, as normal myocardium is used as its reference. ECV using pre-and post-contrast T1 mapping has been validated against histologically proven myocardial fibrosis. This was not included in this analysis. Also, first-pass perfusion imaging for the assessment of myocardial ischemia and parametric T2 mapping were not included in this analysis.

Coronary angiography was performed on clinical indications, as determined by the attending cardiologist, and coronary artery anatomy was not assessed in patients with acute pulmonary edema and neurological emergency.

Due to the high rate of non-adherence to medications and de novo presentation, the history of antihypertensive medication and their relationship with structural and functional cardiac alterations could not be determined with accuracy. The cross-sectional study design limits our ability to determine the prognostic implications of asymmetric hypertrophy and LGE. Larger, longitudinal outcome studies will be required to determine the prognostic implications of our findings. These limitations notwithstanding, our study provides important data, highlighting clinically significant CMR findings among patients with hypertensive crisis, including the prevalence of nonischemic LGE, maximum wall thickness of >15 mm, and asymmetric LVH.

## 5. Conclusions

We observed a high prevalence of LVH, asymmetric wall thickening, systolic dysfunction, and nonischemic LGE among patients presenting with hypertensive crisis in a real-world setting. The high prevalence of nonischemic LGE may have prognostic implications beyond what was reported in hypertensive patients without episodes of crisis, and CMR is likely to assist with the improved evaluation of patients to enhance clinical care ultimately. Larger, longitudinal studies are required to determine the prognostic significance of nonischemic LGE in hypertensive crisis.

## Figures and Tables

**Table 1:** Clinical, laboratory, and cardiovascular magnetic resonance parameters of participants.

Participants Characteristic	All Hypertensive Crisis (n = 82)	Hypertensive Urgency (n = 18)	Hypertensive Emergency (n = 64)	*p*-Value
Clinical				
Age, years	48.5 (13.4)	48.4 (15.2)	48.3 (13.1)	0.972
Male, n (%)	47 (57.3)	5 (27.8)	42 (65.6)	0.010
Body mass index (kg/m^2^)	28 (24–34)	33 (27–38)	27 (23–334)	0.054
Systolic blood pressure, mmHg	217 (28)	216 (26)	217 (29)	0.842
Diastolic blood pressure, mmHg	128 (20)	118 (16)	130 (20)	0.032
Chronic kidney disease, n (%)	17 (22)	1 (6)	16 (25)	0.067
Diabetes mellitus, n (%)	13 (17)	4 (22)	9 (14)	0.290
Dyslipidaemia, n (%)	17 (22)	4 (22)	13 (20)	0.527
Non-adherence to medication, n (%)	38 (46)	6 (33)	32 (50)	0.965
De novo presentation, n (%)	26 (32)	4 (22)	22 (34)	0.965
Laboratory				
Creatinine, µmol/L	105 (84–131)	83 (68–97)	113 (93–181)	<0.001
Platelet count, ×10^9^/L	283 (254–349)	290 (254–359)	283 (252–346)	0.937
Hs cTnT, ng/L	25 (12–127)	11 (7–13)	41 (17–162)	<0.001
NT-proBNP, ng/L	377 (64–1566)	90 (35–302)	528 (113–1939)	0.012
Cardiovascular magnetic resonance imaging parameter				
Indexed LV EDV, mL/m^2^	74 (63–101)	65 (60–71)	86 (66–110)	0.001
Indexed LV ESV, mL/m^2^	32 (22–58)	21 (15–29)	39 (24–69)	<0.001
Indexed LV SV, mL/m^2^	43 (38–48)	43 (39–48)	43 (37–49)	0.703
LV ejection fraction, %	57 (43–66)	67 (60–74)	53 (40–63)	<0.001
LV mass, g	200 (152–273)	156 (130–185)	221 (167–295)	0.001
Indexed LV mass, g/m^2^	107 (83–140)	81 (66–103)	116 (98–153)	<0.001
Maximum LV wall thickness, mm	15.4 (3.2)	13.6 (1.6)	15.3 (2.5)	0.047
LV mass: volume ratio, g/mL	1.3 (1.1–1.6)	1.22 (1.07–1.55)	1.31 (1.10–1.64)	0.445
LV hypertrophy, n (%)	65 (79)	9 (50)	56 (88)	0.002
Indexed RV EDV, mL/m^2^	66 (58–78)	64 (58–69)	70 (58–80)	0.146
Indexed RV ESV, mL/m^2^	24 (19–34)	23 (16–28)	26 (19–36)	0.128
Indexed RV SV, mL/m^2^	40 (35–46)	38 (37–46)	41 (35–46)	0.881
RV ejection fraction, %	62 (56–67)	65 (61–73)	62 (53–67)	0.092
Indexed LA volume, mL/m^2^	40 (30–51)	31 (28–37)	43 (31–55)	0.004
LGE, n/contrast study, n (%)	52/69 (75)	8/18 (44)	44/51 (86)	<0.001
Ischemic pattern	11/69 (16)	0 (0)	11/51 (22)	
Nonischemic pattern	41/69 (59)	8/18 (44)	33/51 (65)	

Abbreviations: hs cTnT, high-sensitivity cardiac troponin T; NT-proBNP, N-terminal prohormone of brain natriuretic peptide; LV, left ventricular; EDV, end-diastolic volume, ESV, end-systolic volume; SV, stroke volume; RV, right ventricular, LA, left atrial; and LGE, late gadolinium enhancement.

**Table 2:** Prevalence of different left ventricular geometric patterns.

Left Ventricular Geometry	Hypertensive Crisis (n = 82)	Hypertensive Urgency (n = 18)	Hypertensive Emergency (n = 64)	*p*-Value
Normal geometry, n (%)	5 (6.1)	3 (16.7)	2 (3.1)	0.034
Concentric remodeling, n (%)	12 (14.6)	6 (33.3)	6 (9.4)	0.011
Concentric hypertrophy, n (%)	43 (52.4)	9 (50)	34 (53.1)	0.815
Eccentric hypertrophy, n (%)	22 (26.8)	0 (0)	22 (34.4)	0.004

**Table 3:** Pattern of late gadolinium enhancement in patients with hypertensive crisis.

	n = 52 (%)
A. Ischemic late gadolinium enhancement:	11 (21.2)
Subendocardial with microvascular obstruction	1 (1.9)
Transmural	1 (1.9)
Transmural, mid wall, and right ventricular insertion point	1 (1.9)
Transmural and right ventricular insertion point	1 (1.9)
Transmural with microvascular obstruction	5 (9.6)
Transmural and subendocardial	1 (1.9)
Focal transmural and right ventricular insertion point	1 (1.9)
B. Nonischemic late gadolinium enhancement:	41 (78.8)
Right ventricular insertion point	13 (25)
Focal and right ventricular insertion point	1 (1.9)
Mid wall and right ventricular insertion point	17 (32.7)
Patchy mid wall	7 (13.5)
Focal, mid wall, and right ventricular insertion point	1 (1.9)
Subepicardial, mid wall, and right ventricular insertion point	1 (1.9)
Subepicardial	1 (1.9)

**Table 4:** Factors associated with nonischemic late gadolinium enhancement among patients with hypertensive crisis in a regression analysis.

Variable	Univariable Regression		Multivariable Regression	
	Unadjusted OR (95% CI)	*p*-Value	Adjusted OR (95% CI)	*p*-Value
Age, years	1.003 (0.963–1.045)	0.881	-	NS
Sex	2.157 (0.659–7.064)	0.240	-	NS
Systolic BP, mmHg	1.019 (0.966–1.042)	0.108	-	NS
Diastolic BP, mmHg	1.028 (0.994–1.062)	0.104	-	NS
Creatinine, µmol/L	1.051 (1.018–1.085)	0.002	1.042 (1.006–1.079)	0.002
Lactate dehydrogenase, U/L	1.003 (0.995–1.011)	0.481	-	NS
hs cTnT, ng/L	1.014 (0.993–1.035)	0.200	-	NS
NT-proBNP, ng/L	1.001 (1.000–1.002)	0.123	-	NS
Indexed LV EDV, mL/m^2^	1.023 (0.992–1.054)	0.145	-	NS
Indexed LV ESV, mL/m^2^	1.028 (0.990–1.066)	0.150	-	NS
LV ejection fraction, %	0.970 (0.922–1.020)	0.238	-	NS
LV mass, g	1.031 (1.010–1.052)	0.004	1.025 (1.005–1.046)	0.016
Indexed LV mass, g/m^2^	1.048 (1.017–1.081)	0.003	-	NS
Indexed LA volume, mL/m^2^	1.086 (1.016–1.161)	0.015	-	NS
LV mass/EDV, g/mL	7.008 (1.052–46.67)	0.044	-	NS
Maximum LVWT, mm	1.461 (1.101–1.938)	0.009	-	NS
Asymmetric LVH	2.240 (0.539–9.037)	0.267	-	NS
LGE	11.79 (2.856–48.64)	<0.001	-	NS

Abbreviations: BP, blood pressure; hs cTnT, high-sensitivity cardiac troponin T; NT-proBNP, N-terminal prohormone of brain natriuretic peptide; LV, left ventricular; EDV, end-diastolic volume, ESV, end-systolic volume; SV, stroke volume; LVWT, left ventricular wall thickness; LVH, left ventricular hypertrophy; RV, right ventricular, LA, left atrial; NS, not significant; LGE, late gadolinium enhancement.

## Data Availability

The authors declare their willingness to share the data for this research upon a reasonable request, and approval by the Stellenbosch University.

## Supplementary Materials

The following supporting information can be downloaded at: https://www.mdpi.com/article/10.3390/jcdd10090367/s1, Table S1: Comparison of cardiac magnetic resonance imaging findings in subtypes hypertensive crisis; Table S2: Comparison of clinical and cardiovascular magnetic resonance profiles of hypertensive crisis patients based on asymmetric left ventricular hypertrophy; Table S3: Comparison of clinical and cardiovascular magnetic resonance profile of hypertensive crisis patients with and without left ventricular hypertrophy; and Table S4: Comparison of clinical and cardiovascular magnetic resonance profile of hypertensive patients with and without nonischemic LGE.

## References

[1] Mchomvu E., Mbunda G., Simon N., Kitila F., Temba Y., Msumba I., Namamba J., Kilindimo S., Mgubike H., Gingo W. (2019). Diagnoses made in an Emergency Department in rural sub-Saharan Africa. Swiss Med. Wkly..

[2] van den Born B.J.H., Lip G.Y.H., Brguljan-Hitij J., Cremer A., Segura J., Morales E., Mahfoud F., Amraoui F., Persu A., Kahan T. (2019). ESC Council on hypertension position document on the management of hypertensive emergencies. Eur. Heart J. Cardiovasc. Pharmacother..

[3] Zampaglione B., Pascale C., Marchisio M., Cavallo-Perin P. (1996). Hypertensive urgencies and emergencies. Prevalence and clinical presentation. Hypertension.

[4] Astarita A., Covella M., Vallelonga F., Cesareo M., Totaro S., Ventre L., Aprà F., Veglio F., Milan A. (2020). Hypertensive emergencies and urgencies in emergency departments: A systematic review and meta-analysis. J. Hypertens..

[5] Tadic M., Cuspidi C., Marwick T.H. (2022). Phenotyping the hypertensive heart. Eur. Heart J..

[6] Peterson G.E., De Backer T., Contreras G., Wang X., Kendrick C., Greene T., Appel L.J., Randall O.S., Lea J., Smogorzewski M. (2013). Relationship of left ventricular hypertrophy and diastolic function with cardiovascular and renal outcomes in African Americans with hypertensive chronic kidney disease. Hypertension.

[7] Garg S., De Lemos J.A., Ayers C., Khouri M.G., Pandey A., Berry J.D., Peshock R.M., Drazner M.H. (2015). Association of a 4-Tiered Classification of LV Hypertrophy with Adverse CV Outcomes in the General Population. JACC Cardiovasc. Imaging.

[8] Messerli F.H. (1999). Hypertension and sudden cardiac death. Am. J. Hypertens..

[9] Chatterjee S., Bavishi C., Sardar P., Agarwal V., Krishnamoorthy P., Grodzicki T., Messerli F.H. (2014). Meta-analysis of left ventricular hypertrophy and sustained arrhythmias. Am. J. Cardiol..

[10] Iyer N.R., Le T.T., Kui M.S.L., Tang H.C., Chin C.T., Phua S.K., Bryant J.A., Pua C.J., Ang B., Toh D.F. (2022). Markers of Focal and Diffuse Nonischemic Myocardial Fibrosis Are Associated With Adverse Cardiac Remodeling and Prognosis in Patients with Hypertension: The REMODEL Study. Hypertension.

[11] Burchell A.E., LRodrigues J.C., Charalambos M., KRatcliffe L.E., Hart E.C., RPaton J.F., Baumbach A., Manghat N.E., Nightingale A.K. (2017). Comprehensive First-Line Magnetic Resonance Imaging in Hypertension: Experience From a Single-Center Tertiary Referral Clinic. J. Clin. Hypertens..

[12] Romano S., Judd R.M., Kim R.J., Kim H.W., Heitner J.F., Shah D.J., Devereux R.B., Salazar P., Trybula M., Chia R.C. (2019). Prognostic Implications of Mitral Annular Plane Systolic Excursion in Patients with Hypertension and a Clinical Indication for Cardiac Magnetic Resonance Imaging: A Multicenter Study. JACC Cardiovasc. Imaging.

[13] Wachtell K., Palmieri V., Gerdts E., Bella J.N., Aurigemma G.P., Papademetriou V., Dahlöf B., Aalto T., Ibsen H., Rokkedal J.E. (2010). Prognostic Significance of Left Ventricular Diastolic Dysfunction in Patients with Left Ventricular Hypertrophy and Systemic Hypertension (the LIFE Study). Am. J. Cardiol..

[14] Gandhi S.K., Powers J.C., Nomeir A.-M., Fowle K., Kitzman D.W., Rankin K.M., Little W.C. (2001). The Pathogenesis of Acute Pulmonary Edema Associated with Hypertension. N. Eng. J. Med..

[15] Gosse P., Coulon P., Papaioannou G., Litalien J., Lemetayer P. (2011). Impact of malignant arterial hypertension on the heart. J. Hypertens..

[16] Grajewski K.G., Stojanovska J., Ibrahim E.S.H., Sayyouh M., Attili A. (2020). Left Ventricular Hypertrophy: Evaluation with Cardiac MRI. Curr. Probl. Diagn. Radiol..

[17] Rodrigues J.C.L., Amadu A.M., Dastidar A.G., Hassan N., Lyen S.M., Lawton C.B., Ratcliffe L.E., Burchell A.E., Hart E.C., Hamilton M.C. (2016). Prevalence and predictors of asymmetric hypertensive heart disease: Insights from cardiac and aortic function with cardiovascular magnetic resonance. Eur. Heart J. Cardiovasc. Imaging.

[18] Kulkarni S., Glover M., Kapil V., Abrams S.M.L., Partridge S., McCormack T., Sever P., Delles C., Wilkinson I.B. (2022). Management of hypertensive crisis: British and Irish Hypertension Society Position document. J. Hum. Hypertens..

[19] Thygesen K., Alpert J.S., Jaffe A.S., Chaitman B.R., Bax J.J., Morrow D.A., White H.D., Executive Group on behalf of the Joint European Society of Cardiology (ESC), American College of Cardiology (ACC), American Heart Association (AHA) (2018). Fourth universal definition of myocardial infarction (2018). J. Am. Coll. Cardiol..

[20] Kramer C.M., Barkhausen J., Bucciarelli-Ducci C., Flamm S.D., Kim R.J., Nagel E. (2020). Standardized cardiovascular magnetic resonance imaging (CMR) protocols: 2020 update. J. Cardiovasc. Magn. Reson..

[21] Almehmadi F., Joncas S.X., Nevis I., Zahrani M., Bokhari M., Stirrat J., Fine N.M., Yee R., White J.A. (2014). Prevalence of myocardial fibrosis patterns in patients with systolic dysfunction: Prognostic significance for the prediction of sudden cardiac arrest or appropriate implantable cardiac defibrillator therapy. Circ. Cardiovasc. Imaging.

[22] Mikami Y., Cornhill A., Dykstra S., Satriano A., Hansen R., Flewitt J., Seib M., Rivest S., Sandonato R., Lydell C.P. (2021). Right ventricular insertion site fibrosis in a dilated cardiomyopathy referral population: Phenotypic associations and value for the prediction of heart failure admission or death. J. Cardiovasc. Magn. Reson..

[23] Kawel-Boehm N., Maceira A., Valsangiacomo-Buechel E.R., Vogel-Claussen J., Turkbey E.B., Williams R., Plein S., Tee M., Eng J., A Bluemke D. (2015). Normal values for cardiovascular magnetic resonance in adults and children. J. Cardiovasc. Magn. Reson..

[24] Buchner S., Debl K., Haimerl J., Djavidani B., Poschenrieder F., Feuerbach S., Riegger G.A., Luchner A. (2009). Electrocardiographic diagnosis of left ventricular hypertrophy in aortic valve disease: Evaluation of ECG criteria by cardiovascular magnetic resonance. J. Cardiovasc. Magn. Reson..

[25] Dweck M.R., Joshi S., Murigu T., Gulati A., Alpendurada F., Jabbour A., Maceira A., Roussin I., Northridge D.B., Kilner P.J. (2012). Left ventricular remodeling and hypertrophy in patients with aortic stenosis: Insights from cardiovascular magnetic resonance. J. Cardiovasc. Magn. Reson..

[26] Ganau A., Devereux R.B., Roman M.J., de Simone G., Pickering T.G., Saba P.S., Vargiu P., Simongini I., Laragh J.H. (1992). Patterns of left ventricular hypertrophy and geometric remodeling in essential hypertension. J. Am. Coll. Cardiol..

[27] Bellenger N.G., Davies L.C., Francis J.M., Coats A.J.S., Pennell D.J. (2000). Reduction in sample size for studies of remodeling in heart failure by the use of cardiovascular magnetic resonance. J. Cardiovasc. Magn. Reson..

[28] Robbertse P.S., Doubell A.F., Steyn J., Lombard C.J., Talle M.A., Herbst P.G. (2023). Altered cardiac structure and function in newly diagnosed people living with HIV: A prospective cardiovascular magnetic resonance study after the initiation of antiretroviral treatment. Int. J. Cardiovasc. Imaging.

[29] Rubin S., Cremer A., Boulestreau R., Rigothier C., Kuntz S., Gosse P. (2019). Malignant hypertension: Diagnosis, treatment and prognosis with experience from the Bordeaux cohort. J. Hypertens..

[30] Vallelonga F., Cesareo M., Menon L., Leone D., Lupia E., Morello F., Totaro S., Aggiusti C., Salvetti M., Ioverno A. (2023). Hypertensive emergencies and urgencies: A preliminary report of the ongoing Italian multicentric study ERIDANO. Hypertens. Res..

[31] Schillaci G., Verdecchia P., Porcellati C., Cuccurullo O., Cosco C., Perticone F. (2000). Continuous relation between left ventricular mass and cardiovascular risk in essential hypertension. Hypertension.

[32] Verdecchia P., Carini G., Circo A., Dovellini E., Giovannini E., Lombardo M., Solinas P., Gorini M., Maggioni A.P., MAVI (MAssa Ventricolare sinistra nell’Ipertensione) Study Group (2001). Left ventricular mass and cardiovascular morbidity in essential hypertension: The MAVI study. J. Am. Coll. Cardiol..

[33] Li T., Li G., Guo X., Li Z., Sun Y. (2021). Echocardiographic left ventricular geometry profiles for prediction of stroke, coronary heart disease and all-cause mortality in the Chinese community: A rural cohort population study. BMC Cardiovasc. Disord..

[34] Mayet J., Shahi M., Poulter N.R., Sever P.S., Foale R.A., Thom S.A.M.G. (1997). Left ventricular geometry in presenting untreated hypertension. J. Hum. Hypertens..

[35] Pattanshetty D.J., Bhat P.K., Aneja A., Pillai D.P. (2012). Elevated troponin predicts long-term adverse cardiovascular outcomes in hypertensive crisis: A retrospective study. J. Hypertens..

[36] Wicker P., Roudaut R., Haissaguere M., Villega-Arino P., Clementy J., Dallocchio M. (1983). Prevalence and significance of asymmetric septal hypertrophy in hypertension: An echocardiographic and clinical study. Eur. Heart J..

[37] Maron B.J., Edwards J.E., Epstein S.E. (1978). Disproportionate ventricular septal thickening in patients with systemic hypertension. Chest.

[38] Suzuki J., Matsubara H., Urakami M., Inada M. (1993). Rat angiotensin II (type 1A) receptor mRNA regulation and subtype expression in myocardial growth and hypertrophy. Circ. Res..

[39] Ostman-Smith I. (1981). Cardiac sympathetic nerves as the final common pathway in the induction of adaptive cardiac hypertrophy. Clin. Sci..

[40] Rudolph A., Abdel-Aty H., Bohl S., Boyé P., Zagrosek A., Dietz R., Schulz-Menger J. (2009). Noninvasive detection of fibrosis applying contrast-enhanced cardiac magnetic resonance in different forms of left ventricular hypertrophy relation to remodeling. J. Am. Coll. Cardiol..

[41] Treibel T.A., Zemrak F., Sado D.M., Banypersad S.M., White S.K., Maestrini V., Barison A., Patel V., Herrey A.S., Davies C. (2015). Extracellular volume quantification in isolated hypertension—Changes at the detectable limits?. J. Cardiovasc. Magn. Reson..

[42] Hwang J.W., Lee S.C., Kim D., Kim J., Kim E.K., Chang S.A., Park S.J., Kim S.M., Choe Y.H., Park S.W. (2023). Role of cardiovascular magnetic resonance imaging and cardiopulmonary exercise test in predicting composite clinical outcomes in patients with hypertrophic cardiomyopathy. PLoS ONE.

[43] Kuribayashi T., Roberts W.C. (1992). Myocardial disarray at junction of ventricular septum and left and right ventricular free walls in hypertrophic cardiomyopathy. Am. J. Cardiol..

[44] Andersen S., Nielsen-Kudsk J.E., Vonk Noordegraaf A., De Man F.S. (2019). Right Ventricular Fibrosis. Circulation.

