# Supplementary material for: Cardiac Morphology, Function, and Left Ventricular Geometric Pattern in Patients with Hypertensive Crisis: A Cardiovascular Magnetic Resonance-Based Study
Source: J Cardiovasc Dev Dis. 2023 Aug 27;10(9):367. doi: 10.3390/jcdd10090367 (PMC10532285; doi:10.3390/jcdd10090367)
Supplement: Supplementary file 1 [file jcdd-10-00367-s001.zip › jcdd-2546399-supplementary.pdf]

Supplemental Table S1: Comparison of cardiac magnetic resonance imaging findings in subtypes hypertensive crisis.

Variable	Hypertensive crisis (n=82)				*P* value
	Hypertensive urgency (n=18)	Acute pulmonary oedema (n=22)	Myocardial infarction (n=20)	Neurological emergencies (n=22)	
Age, years	48.4 (15.2)	49.1 (14.3)	54.2 (12.7)	43.1 (9.7)	0.098
Male, n(%)	5 (27.8)	14 (63.6)	13 (65)	15 (68.2)	0.045
Systolic BP (mmHg)	215.9 (26)	225.6 (27.3)	202 (25.6)	223.9 (29.1)	0.036*
Diastolic BP (mmHg)	118.1 (16.1)	136.6 (19.1)	117 (17.1)	136 (18.6)	<0.001[abcd]
Indexed LV EDV, mL/m²	64.8 [60–71]	109 [91-140]	73.4 [67–98]	66.4 [59– 88]	<0.001[ae]
Indexed LV ESV, mL/m²	20.7 [15–29]	69.7 [54–86]	32.6 [23–53]	28.9 [21–38]	<0.001[abef]
Indexed LV SV, mL/m²	42.6 [39–48]	42.9 [35–53]	43.9 [41–48]	40.7 [33–45]	0.628
LV ejection fraction, %	67 [60–74]	38.5 [33–43]	58 [45–65]	58 [54–66]	<0.001[abe]
LV mass, g	156 [130–185]	255 [199–299]	187.5 [159–257]	216 [146–316]	0.001[ac]
Indexed LV mass, g/m²	81.3 [66–103]	133.3 [109–166]	103.9 [82–134]	110 [85–148]	<0.001[ac]
Max wall thickness (mm)	13.6 (1.6)	16 (2.4)	14.4 (2.3)	17 (4.4)	<0.001[acd]
LV mass: volume ratio (g/mL)	1.22 [1.07–1.55]	1.25 [1.04–1.53]	1.17 [1.04–1.59]	1.54 [1.29–1.87]	0.016[e]
LV hypertrophy, n (%)	9 (50)	22 (100)	15 (75)	19 (86.4)	<0.001
Indexed RV EDV, mL/m²	64.3 [58–67]	75.3 [59–95]	71 [59–79]	62.8 [54–74]	0.167
Indexed RV ESV, mL/m²	22.7 [16– 28]	32 [20–46]	24 [19–32]	22.3 [18–32]	0.154
Indexed RV SV, mL/m²	38.3 [37–46]	41 [32–50]	41.5 [37–46]	40.3 [33–42]	0.810
RV ejection fraction, %	64.5 [61–73]	60 [47–64]	62 [58–69]	63.2 [54–68]	0.104
Indexed LA volume, mL/m²	31 [28–37]	54.2 [40–62]	43.3 [35–50]	32.3 [27–44]	<0.001[aef]
LGE, n(%)	8/18 (44.4)	11/12 (91.7)	16/18 (88.9)	16/20 (80)	0.008

LV, left ventricular; EDV, end-diastolic volume, ESV, end-systolic volume; SV, stroke volume; RV, right ventricular, LA, left atrial; LGE, late gadolinium enhancement. **P > 0.05 on multiple comparison using Bonferroni Correction; [a]P <0.05 for acute pulmonary oedema versus hypertensive urgency; [b]P <0.05 for acute pulmonary oedema versus myocardial infarction; [c]P <0.05 for neurological emergencies versus hypertensive urgency; [d]P <0.05 for neurological emergencies versus myocardial infarction; [e]P <0.05 for acute pulmonary oedema versus neurological emergencies; [f]P <0.05 for myocardial infarction versus hypertensive urgency.*

Supplemental Table S2: Comparison of clinical and cardiovascular magnetic resonance profiles of hypertensive crisis patients based on asymmetric left ventricular hypertrophy.

Participants characteristics	All hypertensive crisis (n=58)	LVH without asymmetry (n=45)	Asymmetric LVH (n=13)	*P*
Clinical				
Age, years	47.8 (14)	48.5 (14)	45.6 (14)	0.688
Male, n(%)	30 (52)	22 (49)	8 (62)	0.534
Body mass index (kg/m^2)	29 [24–36]	29 [24–37]	28 [23–37]	0.695
Systolic BP (mmHg)	217 (28)	215 (27)	227 (29)	0.198
Diastolic BP(mmHg)	128 (20)	126 (19)	135 (19)	0.140
Laboratory				
Creatinine (μmol/L)	98 [81–116]	93 [80–116]	108 [82–125]	0.386
Platelet count (x 10^9/L)	285 [257–341]	295 [258–341]	284 [255–398]	0.590
hs cTnT (ng/L)	17 [11–36]	16 [8–36]	20 [14–47]	0.165
NT-proBNP (ng/L)	208 [38–783]	189 [40–838]	275 [41–932]	0.618
Cardiac magnetic resonance Parameters				
Indexed LV EDV, mL/m^2	68 [62–91]	70 [62–89]	68 [57–112]	0.244
Indexed LV ESV, mL/m^2	29 [21–45]	28 [21–48]	29 [19–58]	0.933
Indexed LV SV, mL/m^2	43 [38–48]	44 [37–48]	40 [36–51]	0.073
LV ejection fraction, %	60 [51–67]	60 [51–68]	58 [46–66]	0.688
LV mass, g	188 [148–265]	172 [147–239]	261 [146–312]	0.014
Indexed LV mass, g/m^2	104 [80–129]	98 [77–123]	119 [95–174]	0.019
Maximum LV wall thickness (mm)	15.6 (3.4)	14.3 (2.4)	19 (3.3)	<0.001
LV mass: volume ratio (g/mL)	1.35 [1.1–1.64]	1.18 [1.1–1.59]	1.54 [1.16–1.92]	0.005
Indexed RV EDV, mL/m^2	65 [57–75]	66 [60–76]	61 [51–87]	0.259
Indexed RV ESV, mL/m^2	23 [18–33]	24 [19–33]	23 [16–39]	0.376
Indexed RV SV, mL/m^2	39 [35–45]	41 [36–46]	38 [33–46]	0.215
RV ejection fraction, %	63 [58–72]	63 [58–70]	62 [48–72]	0.520
Indexed LA volume, mL/m^2	33 [30–47]	34 [30–47]	36 [25–47]	0.218
LGE, n(%)	41 (71)	29 (64)	12 (92)	0.082

BP, blood pressure; hs cTnT, high sensitivity cardiac troponin T; NT-proBNP, N-terminal prohormone of brain natriuretic peptide; LV, left ventricular; EDV, end-diastolic volume, ESV, end-systolic volume; SV, stroke volume; RV, right ventricular, LA, left atrial; LGE, late gadolinium enhancement.

Supplemental Table S3: Comparison of clinical and cardiovascular magnetic resonance profile of hypertensive crisis patients with and without left ventricular hypertrophy.

Participant characteristics	Hypertensive crisis (n=82)	LVH (n=64)	No LVH (n=18)	*P*
Clinical				
Age, years	48.5 (13.4)	48.9 (14.2)	47 (9.9)	0.680
Male, n(%)	47 (57.3)	36 (55.4)	11 (64.7)	0.587
Body mass index (kg/m^2)	27.9 [24–34]	26.8 [23– 34]	30.9 [28–38]	0.20
Systolic blood pressure (mmHg)	217.2 (28.3)	222.3 (26.7)	197.8 (26.3)	0.002
Diastolic blood pressure(mmHg)	127.6 (19.9)	131.1 (20.4)	113.9 (9.9)	<0.001
Laboratory				
Creatinine (micromol/L)	104.5 [84–131]	111 [88–174]	85 [77–102]	0.002
Platelet count (x 10^9/L)	283 [254–349]	283 [253–335]	286 [249–361]	0.945
hs-cTnT (ng/L)	25 [12–127]	35.5 [16–152]	8 [6–19]	<0.001
NT-proBNP (ng/L)	377 [64–1566]	534 [180–2077]	32 [19–87]	<0.001
Cardiac magnetic resonance parameters				
Indexed LV EDV, mL/m^2	73.7 [63–101]	86.1 [66–109]	64.2 [60–70]	<0.001
Indexed LV ESV, mL/m^2	32.2 [22–58]	37.9 [24–69]	22.2 [18–28]	<0.001
Indexed LV SV, mL/m^2	43.1 [38–48]	43.3 [38–49]	42.5 [37–46]	0.668
LV ejection fraction, %	57 [42–66]	53 [40–65]	64 [59–71]	<0.001
LV mass, g	200 [152–273]	225 [173–297]	147 [126–155]	<0.001
Indexed LV mass, g/m^2	106.7 [83–140]	117.9 [101–153]	69.4 [62–77]	<0.001
Maximum LV wall thickness (mm)	15.4 (3.2)	15.9 (3.3)	13.3 (1.7)	<0.001
LV mass: volume ratio (g/mL)	1.3 [1.1–1.6]	1.42 [1.13–1.67]	1.1 [1.0–1.45]	<0.001
Indexed RV EDV, mL/m^2	65.9 [58–78]	66.2 [57–79]	64.9 [60–73]	0.680
Indexed RV ESV, mL/m^2	24.1 [19–34]	23.6 [18–36]	26.9 [20–32]	0.680
Indexed RV SV, mL/m^2	40.4 [35–46]	40.4 [35–47]	38.5 [36–45]	0.740
RV ejection fraction, %	62 [56–67]	62 [54–70]	61 [59–66]	0.423
Indexed LA volume, mL/m^2	40.3 [30–51]	43.8 [31–55]	30.3 [29–34]	0.001
LGE, n(%)	52/69 (75.4)	44/51 (86.3)	7/17 (41.2)	<0.001

hscTnT, high sensitivity cardiac troponin T; NT-proBNP, N-terminal prohormone of brain natriuretic peptide; LV, left ventricular; EDV, end-diastolic volume, ESV, end-systolic volume; SV, stroke volume; ASH, asymmetric septal hypertrophy; RV, right ventricular, LA, left atrial; LGE, late gadolinium enhancement.

Supplemental Table S4: Comparison of clinical and cardiovascular magnetic resonance profile of hypertensive crisis patients with and without nonischaemic LGE.

Participant characteristics	Hypertensive crisis (n=58)	No LGE (n=17)	Nonischaemic LGE (n=41)	*P*
Clinical				
Age, years	47.8 (14)	47.4 (14.2)	48 (14.1)	0.817
Male, n(%)	30 (51.7)	6 (35.3)	24 (58.5)	0.151
Body mass index (kg/m^2)	29 [24–36]	27.9 [22–37]	29 [25–37]	0.343
Systolic BP (mmHg)	217.7 (27.4)	207.2 (22.7)	222 (28.4)	0.092
Diastolic BP (mmHg)	128.2 (19.5)	121.5 (18)	130.9 (20)	0.067
Laboratory				
Creatinine (mol/L)	98 [81 –116]	77 [60–92]	108 [88–119]	<0.001
Platelet count (x 10^9/L)	285 [257–341]	304 [263–369]	283.5 [256–318]	0.295
hs cTnT (ng/L)	17 [11–36]	7.5 [5–17]	21 [13–39]	0.001
NT-proBNP (ng/L)	208 [38–783]	51 [15–151]	396 [113–1037]	0.001
Cardiac magnetic resonance				
Indexed LV EDV, mL/m^2	68.4 [62–91]	61.8 [58–70]	73.5 [63–100]	0.009
Indexed LV ESV, mL/m^2	28.6 [21–45]	23.1 [17–31]	30.8 [21– 53]	0.069
Indexed LV SV, mL/m^2	42.7 [38–48]	39.3 [35–46]	43.6 [38–53]	0.146
LV ejection fraction, %	59.5 [51–67]	61 [55–71]	58 [45–67]	0.191
LV mass, g	188 [148–265]	147 [124–170]	207 [165–299]	<0.001
Indexed LV mass, g/m^2	104.3 [80–129]	75.2 [62–93]	111 [96–142]	<0.001
Maximum LV wall thickness (mm)	15.5 (3.4)	13.5 (1.8)	16.4 (3.6)	0.001
LV mass: volume ratio (g/mL)	1.35 [1.10–1.64]	1.13 [1.0–1.57]	1.43 [1.11–1.74]	0.043
LV hypertrophy, n(%)	44 (75.9)	7 (41.2)	37 (90.2)	<0.001
Indexed RV EDV, mL/m^2	65.2 [57–75]	63.9 [54–68]	66 [58–79]	0.228
Indexed RV ESV, mL/m^2	22.7 [18–33]	22.5 [19–31]	22.9 [16–34]	0.986
Indexed RV SV, mL/m^2	38.5 [35–45]	37 [30–43]	40.4 [36–46]	0.169
RV ejection fraction, %	63 [58–72]	62 [57–66]	64 [58–73]	0.393
Indexed LA volume, mL/m^2	33.4 [30–47]	29.7 [26–34]	40.4 [30–52]	0.005

BP, blood pressure; hs cTnT, high sensitivity cardiac troponin T; NT-proBNP, N-terminal prohormone of brain natriuretic peptide; LV, left ventricular; LVH, left ventricular hypertrophy; EDV, end-diastolic volume, ESV, end-systolic volume; SV, stroke volume; RV, right ventricular, LA, left atrial; LGE, late gadolinium enhancement.
